# The association of opening K–12 schools with the spread of COVID-19 in the United States: County-level panel data analysis

**DOI:** 10.1073/pnas.2103420118

**Published:** 2021-10-12

**Authors:** Victor Chernozhukov, Hiroyuki Kasahara, Paul Schrimpf

**Affiliations:** ^a^Department of Economics, Massachusetts Institute of Technology, Cambridge, MA 02142;; ^b^Center for Statistics and Data Science, Massachusetts Institute of Technology, Cambridge, MA 02139;; ^c^Vancouver School of Economics, University of British Columbia, Vancouver, BC V6T1L4, Canada

**Keywords:** K–12 school openings, in-person, hybrid, and remote, mask-wearing requirements for staff, foot traffic data, debiased estimator

## Abstract

This paper empirically examines how the opening of K–12 schools is associated with the spread of COVID-19 using county-level panel data in the United States. As preliminary evidence, our event-study analysis indicates that cases and deaths in counties with in-person or hybrid opening relative to those with remote opening substantially increased after the school opening date, especially for counties without any mask mandate for staff. Our main analysis uses a dynamic panel data model for case and death growth rates, where we control for dynamically evolving mitigation policies, past infection levels, and additive county-level and state-week “fixed” effects. This analysis shows that an increase in visits to both K–12 schools and colleges is associated with a subsequent increase in case and death growth rates. The estimates indicate that fully opening K–12 schools with in-person learning is associated with a 5 (SE = 2) percentage points increase in the growth rate of cases. We also find that the association of K–12 school visits or in-person school openings with case growth is stronger for counties that do not require staff to wear masks at schools. These findings support policies that promote masking and other precautionary measures at schools and giving vaccine priority to education workers.

## Significance

This paper examines whether the opening of K–12 schools may lead to the spread of COVID-19. Analyzing how an increase of COVID-19 cases is related to the timing of opening K–12 schools in the United States, we find that counties that opened K–12 schools with in-person learning experienced an increase in the growth rate of cases by 5 percentage points on average, controlling for a variety of policies, past infection rates, and other factors. This association of K–12 school visits with case growth is stronger when mask wearing is not mandated for staff at school. These findings support policies that promote masking and other precautionary measures at schools and giving vaccine priority to education workers.

Does opening K–12 schools lead to the spread of COVID-19? Do mitigation strategies such as mask-wearing requirements help reduce the transmission of severe acute respiratory syndrome coronavirus 2 (SARS-CoV-2) at school? These are important policy-relevant questions in countries with low vaccination rates, especially given the emerging variants of concern with higher transmission rates. If in-person school openings substantially increase COVID-19 cases, then local governments could promote mitigation measures at schools (universal and proper masking, social distancing, and handwashing) to lower the risk of COVID-19 spread. Furthermore, the governments could prioritize vaccines for education workers and elderly parents in the case of in-person school openings. This paper uses county-level panel data on K–12 school opening plans and mitigation strategies together with foot traffic data to investigate how an increase in visits to K–12 schools is associated with a subsequent increase in COVID-19 cases in the United States.

## Data

We begin with describing our data and provide descriptive evidence. Our sample period is from 1 April 2020 to 2 December 2020. Our analysis uses county-level panel data in the United States. As outcome variables, we use weekly cases and deaths as well as their growth rates. The main explanatory variables of interest are school openings with different teaching methods and mitigation measures from MCH Strategic Data and per-device visits to K–12 schools from SafeGraph foot traffic data. We also use the foot traffic data on stay-at-home devices and visits to full-time/part-time workplaces, colleges/universities, restaurants, bars, recreational facilities, and churches. Our panel regression analysis uses additional data on nonpharmaceutical policy interventions (NPIs) and the number of tests.

The data on cases and deaths for each county are from *The New York Times* (NYT) (1). SafeGraph provides foot traffic data based on a panel of global positioning system pings from anonymous mobile devices. Per-device visits to K–12 schools, colleges/universities, restaurants, bars, recreational places, and churches are constructed from the ratio of daily device visits to these point-of-interest locations to the number of devices residing in each county. Full-time and part-time workplace visits are the ratio of the number of devices that spent more than 6 h and between 3 and 6 h, respectively, at one location other than one’s home location to the total number of device counts. The staying-home device variable is the ratio of the number of devices that do not leave home locations to the total number of device counts.

MCH Strategic Data (2) provides information on the date of school openings with different teaching methods (in person, hybrid, and remote) as well as mitigation strategies at 14,703 school districts. We link school district-level MCH data to the county-level data from NYT and SafeGraph using the file for School Districts and Associated Counties from the US Census Bureau. School district data are aggregated up to county level using the enrollment of students at each district. Specifically, we construct the proportion of students with different teaching methods for each county-day observation using the district-level information on school opening dates and teaching methods. We define each teaching method’s county-level school opening date by the weighted mean of district-level school opening dates of the corresponding teaching method with enrollment weights. We also construct a county-level dummy variable of “no mask requirement for staff,” which takes a value of 1 if there exists at least one school district with no mask requirement. The measure of mask requirements for staff is highly correlated with other mitigation measures, including mask requirements for students, prohibiting sports activities, and online instruction increases as shown in *SI Appendix*, Table S3.^*^ A substantial fraction of school districts report “unknown” or “pending” for teaching methods and mask requirements. We drop county observations from the sample if more than 50% of students attend school districts that report unknown or pending teaching methods or mask requirements for panel regression analysis with teaching methods or mask requirements.

Our empirical analysis uses 7-d moving averages of daily variables to deal with periodic fluctuations within 1 wk. *SI Appendix*, Fig. S4 shows the evolution of percentiles of these variables over time, while *SI Appendix*, Tables S1 and S2 present descriptive statistics and correlation matrix across variables we use for our regression analysis. The dataset contains the maximum of 3,144 counties for regression analysis using foot traffic data but some observations are dropped out of samples due to missing values for school opening teaching methods and staff mask requirements in some specifications.^†^ The analysis was conducted using R software (version 4.0.3).

Table 1 reports the means for the growth rate of weekly confirmed cases and deaths measured by the log difference over 7 d in reported weekly cases/deaths, where the log of weekly cases and deaths is set to be –1 when we observe zero weekly cases and deaths; weekly cases and deaths per 1,000; and per-device visits to K–12 schools, workplaces, and restaurants by teaching methods and separately for periods before and after K–12 school openings. SEs for the means that are two-way clustered on county and date are reported in parentheses. Here and in the event-study analysis, we classify counties into three groups (in person, hybrid, and remote) by the dominant teaching method under which the highest proportion of students are learning within a county.

**Table 1:** Summary statistics

	Wkly case	Wkly death	Wkly cases	Wkly deaths	K–12 sch.	Workplace	Restaurant
	growth	growth	per 1,000	per 1,000	visits	visits	visits
In person							
Before opening							
Mean	0.091	0.013	0.571	0.060	0.045	0.047	0.185
	(0.011)	(0.003)	(0.031)	(0.003)	(0.002)	(0.001)	(0.007)
*N*	52,258	52,258	54,995	54,995	67,070	67,070	67,070
After opening							
Mean	0.143	0.034	3.038	0.104	0.161	0.073	0.188
	(0.014)	(0.006)	(0.200)	(0.005)	(0.005)	(0.001)	(0.006)
*N*	45,749	45,749	45,827	45,827	46,030	46,030	46,030
Difference in means	0.052	0.021	2.467	0.044	0.116	0.026	0.003
	(0.018)	(0.007)	(0.203)	(0.004)	(0.004)	(0.001)	(0.004)
Hybrid							
Before opening							
Mean	0.096	0.024	0.664	0.035	0.036	0.045	0.242
	(0.012)	(0.006)	(0.031)	(0.001)	(0.001)	(0.0004)	(0.005)
*N*	234,820	234,820	243,321	243,321	260,573	260,551	260,573
After opening							
Mean	0.121	0.042	2.368	0.057	0.126	0.064	0.249
	(0.012)	(0.006)	(0.132)	(0.002)	(0.003)	(0.001)	(0.004)
*N*	166,605	166,605	166,660	166,660	167,206	167,206	167,206
Difference in means	0.025	0.019	1.703	0.022	0.090	0.019	0.007
	(0.016)	(0.008)	(0.136)	(0.002)	(0.003)	(0.001)	(0.004)
Remote							
Before opening							
Mean	0.099	0.035	0.742	0.032	0.032	0.045	0.278
	(0.012)	(0.008)	(0.035)	(0.002)	(0.001)	(0.0004)	(0.008)
*N*	76,796	76,796	78,581	78,581	82,165	82,165	82,165
After opening							
Mean	0.103	0.033	1.944	0.047	0.088	0.058	0.287
	(0.012)	(0.009)	(0.115)	(0.003)	(0.003)	(0.001)	(0.007)
*N*	50,127	50,127	50,048	50,048	50,183	50,183	50,183
Difference in means	0.004	–0.002	1.202	0.015	0.056	0.013	0.009
	(0.017)	(0.012)	(0.120)	(0.002)	(0.003)	(0.001)	(0.006)

Statistics are based on observations from 15 April 2020 to 2 December 2020. SEs that are two-way clustered on county and date are reported in parentheses. Wkly, weekly; sch., school.

The school opening dates spread from the beginning of August to late September across counties, where hybrid teaching is more common than remote or in-person teaching (*SI Appendix*, Fig. S4*K*). Reflecting the steady increase in cases from late September to November of 2020 in the United States (*SI Appendix*, Fig. S4*B*), the growth rates of cases and deaths, as well as the number of weekly confirmed cases and deaths, are higher in the period after the school opening compared to the period before. As shown in Table 1, this rise in cases and deaths after the school opening is more pronounced in the counties with in-person or hybrid teaching than in those with remote teaching. K–12 school and workplace visits are also higher after the school openings than before, especially for counties with in-person and hybrid school openings. On the other hand, mean per-device restaurant visits do not change much before and after the school opening, regardless of teaching methods.

**Fig. 1:**
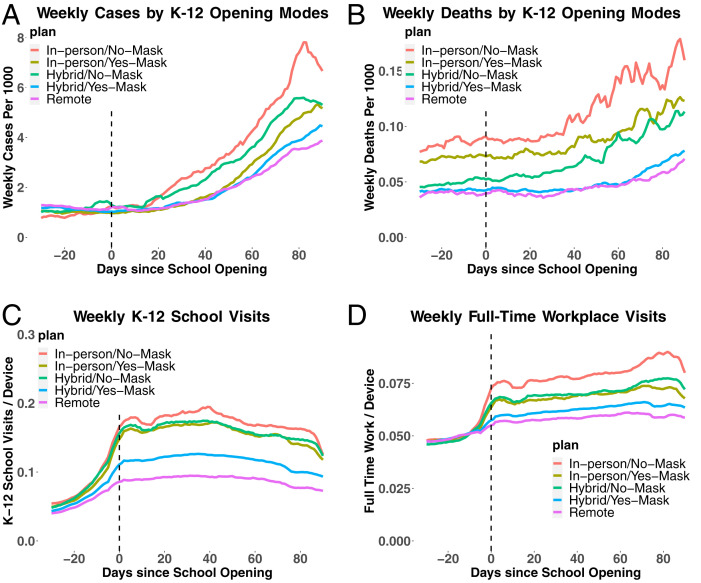
The evolution of cases, deaths, and visits to K–12 schools and restaurants before and after the opening of K–12 schools. *A* and *B* plot the evolution of weekly cases or deaths per 1,000 persons averaged across counties within each group of counties classified by K–12 school teaching methods and mitigation strategy of mask requirements against the days since K–12 school opening. We classify counties that implement in-person teaching as their dominant teaching method into “in-person/yes-mask” and “in-person/no-mask” based on whether at least one school district requires staff to wear masks or not. Similarly, we classify counties that implement hybrid teaching into “hybrid/yes-mask” and “hybrid/no-mask” based on whether mask-wearing is required for staff. We classify counties that implement remote teaching as “Remote.” *C* and *D* plot the evolution of the 7-d average of per-device visits to K–12 schools and full-time workplaces, respectively, against the days since K–12 school opening using the same classification as in *A* and *B*.

Fig. 1 provides visual evidence for the association of opening K–12 schools with the spread of COVID-19 as well as the role of school mitigation strategies. Fig. 1 *A* and *B* plots the evolution of average weekly cases and deaths per 1,000 persons, respectively, against days since school opening for different teaching methods and mask requirements for staff. In Fig. 1*A*, the average number of weekly cases starts increasing after 2 wk of opening schools in person or hybrid for counties with no mask mandates for staff, possibly suggesting that mask mandates at school reduce the transmissions of SARS-CoV-2. In Fig. 1*B*, the number of deaths starts rapidly increasing after 3 to 5 wk of opening schools for counties that adopt in-person/hybrid teaching methods with no mask mandates. Alternative mitigation strategies of requiring mask wearing for the student, prohibiting sports activities, and promoting online instruction also appear to help reduce the number of cases after school openings (*SI Appendix*, Fig. S5 *I–P*).

**Fig. 2:**
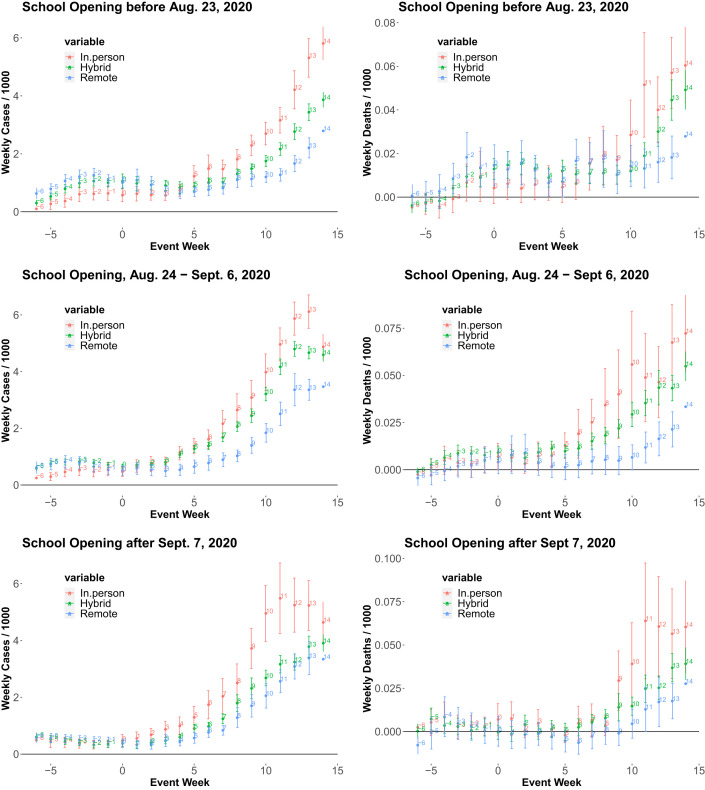
The event-study regression estimates before and after the school opening. Plots show the estimated coefficients for weekly dummies of leads and lags in regression specification **1** with 95% confidence intervals for three subsample periods.

Fig. 1*C* shows that opening K–12 schools in person or hybrid increases the 7-d averages of per-device visits to K–12 schools more than opening remotely, especially when no mask mandates are in place. Fig. 1*D* and *SI Appendix*, Fig. S5 *E* and *F* show that visits to full-time and part-time workplaces increase after school openings with in-person teaching, suggesting that the opening of schools allows parents to return to work.^‡^ On the other hand, we observe no drastic changes in per-device visits to restaurants, recreational facilities, and churches after school openings (*SI Appendix*, Fig. S5 *B–D*).

## A Preliminary Event-Study Analysis

As a matter of preliminary data analysis, we further investigate how cases and deaths change over time after school openings by an “event-study” analysis (e.g., refs. [3]– [5]). We divide the sample into three subsamples, where each subsample contains the observation with similar school opening dates. Then, for each of the subsamples, we run the following regression with weekly dummies of leads and lags for three school opening modes (i.e., in person, hybrid, and remote) with county fixed effects but without time fixed effects:

(1) $$Y_{it}=\sum_{p\in P}\sum_{w=-8}^{22}\gamma_{w}^{p}D_{\tau ,it}^{p}+\alpha_{i}+\epsilon_{it},$$

where *Y_it_* is the number of weekly confirmed cases/deaths per 1,000, $D_{\tau ,it}^{p}$ takes the value equal to 1 if school has been open for *τ* weeks (or will be open after −τ weeks if τ<0) with teaching method p∈P≔{in-person,hybrid,remote} in county *i* at day *t*. The *α_i_* represents a county-specific baseline mean from the beginning of the sample to the 9 wk before the school opening. We consider the event window of 8 wk before the school opening and a maximum of 22 wk after the school opening, where the lag windows are different across subsamples given that our sample ends on 2 December 2020.

Fig. 2 graphs the estimated coefficients over time with 95% confidence intervals with SEs clustered by counties: The first subsample uses county observations that opened schools before 23 August 2020, the second one consists of the counties that opened schools between 24 August and 6 September, and the third one uses the counties with school opening dates after 7 September 2020. The results illustrate that the gap in weekly cases/deaths per 1,000 between remote opening and full/hybrid opening grows over time after the school opening date for all three subsamples.

We also estimate the time-varying predictive effect of in-person or hybrid school openings, as well as that of no mask mandates for staff, relative to remote openings by the estimation method of ref. [3] using their did R package. We define a group by a set of counties with the same school opening date and then estimate the group-time specific average predictive effect of in-person or hybrid opening using the “never-exposed units” and “not-yet-exposed units” as the controls while excluding the “already-exposed units” from the control group. Here, we take the counties with the remote opening plan as the never-exposed-units.^§^ Because almost all counties opened their schools by late September, the estimated predictive effect for the lags primarily reflects the predictive effect of in-person or hybrid openings relative to the counties with remote openings rather than the counties that had not opened schools yet. We report estimates for the average of the group-time specific average predictive effects of in-person or hybrid opening against remote opening across groups with different school opening dates. The predictive effects can be causally interpretable as “exposure (treatment) effect” for the exposed group under the group-by-group parallel trend assumption [3] on counterfactual outcomes in the unexposed state (importantly, please see the discussion below for limitations of interpreting such effects as “actionable”).

**Fig. 3:**
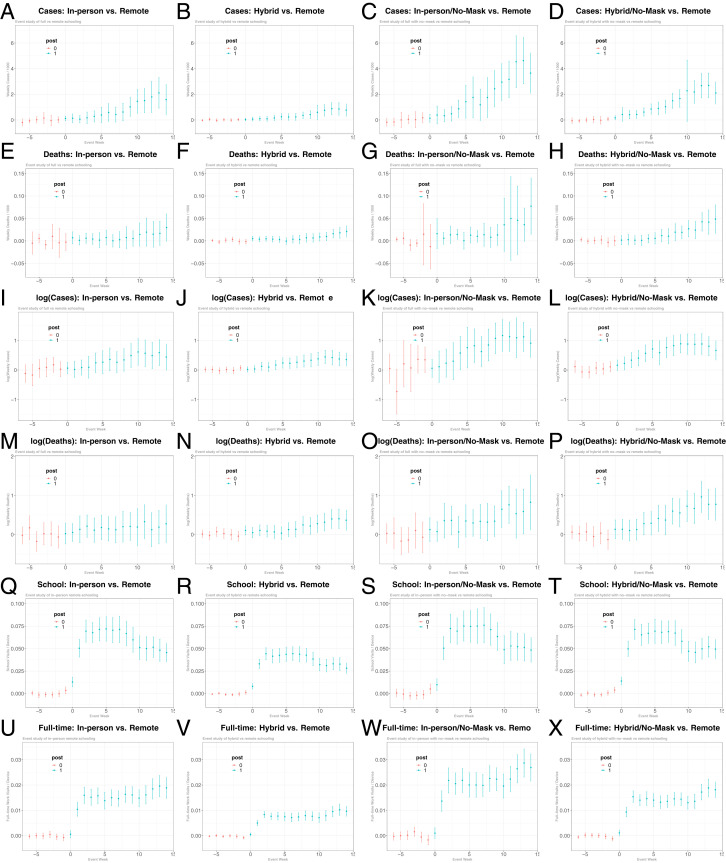
The average treatment estimates obtained using the difference-in-differences (DID) method from Callaway and Sant’Anna [3]. *A* plots the estimates and 95% simultaneous confidence intervals of the average dynamic treatment effect of in-person openings relative to the counties with remote openings as well as the counties that have not opened yet on cases per 1,000 using a subset of counties with either in-person opening or remote opening, where we use the estimation method of ref. [3] implemented by their did R package. Similarly, *B–D* plot the estimates of the average dynamic treatment effect of school opening with hybrid, in-person/mask mandates and hybrid/mask mandates teaching methods, respectively, using a subset of counties with the corresponding teaching method as well as remote opening. *E*–*H*, *I*–*L*, *M*–*P*, *Q*–*T*, and *U*–*X* report the estimates of the average dynamic treatment effect on deaths per 1,000, log(cases), log(deaths), per-device visits to K–12 schools, and per-device visits to full-time workplaces, respectively.

Fig. 3 presents the estimated group-time average predictive effects with 95% simultaneous confidence intervals. Fig. 3 *A–H* shows that cases and deaths in counties with in-person or hybrid openings relative to those with remote openings increase after school openings; furthermore, these increases are more pronounced for counties without any mask mandate for staff in Fig. 3 *C*, *D*, *G*, and *H*. Fig. 3 *I–P* similarly indicates that the log of weekly cases and deaths in counties with in-person or hybrid openings gradually increases after the school opening date, especially for counties with no mask mandates. In Fig. 3 *I–P*, the estimates in the preexposure period are flat and not statistically different from zero, consistent with parallel trend assumption.

**Fig. 4:**
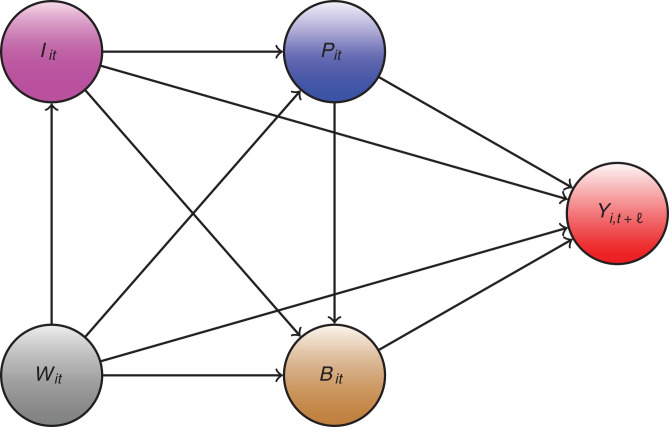
The causal path diagram for our model.

Consistent with the findings in Fig. 1 *C* and *D*, Fig. 3 *Q–X* shows that visits to K–12 schools and full-time workplaces increase after the opening of schools in counties with in-person/hybrid teaching relative to those with remote teaching. In contrast, *SI Appendix*, Fig. S5 indicates no evidence for the association of the school opening date with visits to restaurants, bars, recreational facilities, and churches, suggesting that other unobserved county-level confounders that affect people’s mobilities to these places (e.g., lockdown policies) may not be systematically related to the timing of school opening, teaching modes, and mitigation measures.

Our finding is consistent with that in ref. [6], which examines the data from a massive online survey and finds the association between in-person schooling and COVID-19–related outcomes across counties and the importance of school-based mitigation measures for reducing transmission risks in the United States. In contrast, using an event-study design, refs. [7] and [8] find no evidence that fully opening schools increased case number within the 3 to 4 wk of school openings in Germany. One possible reason for these contradictory findings is that the mitigation measures in German schools may have been more effective in containing in-school transmissions than the measures adopted by the US schools with in-person openings. Another important source of the difference is that the event window length of 3 to 4 wk in refs. [7] and [8] may be too short to identify the effect of school openings on the confirmed cases because asymptomatic, undetected cases are prevalent among children [9].

While our event-study analysis provides valuable preliminary evidence on the predictive links between school reopenings and subsequent increases in infection rates, there are several important limitations in terms of assumptions and interpretation. First, the parallel trend assumption required for causal interpretation of the predictive results might be at odds with the implications of the main epidemiological models that the spread of cases is highly nonlinear [10], with the current number of cases dynamically depending on the number of past infected individuals. Second, the transmission itself is influenced by other containment policies and people’s voluntary behavioral changes in response to information about infection levels [11]. If other mitigation policies respond to past infection levels, the predictive effects derived from the event-study analysis may not capture the “direct causal” effects (i.e., effects that hold other policies and other state variables fixed) of the target school-reopening policies, but rather the “total effect” for the exposed group. This makes it difficult to generate actionable policy insights. For instance, in an extreme case, the total effect may be zero because other mitigation policies completely offset the effect of the target policy. Therefore, in such cases, one may mistakenly conclude that the target school-reopening policy is “safe” and should be implemented regardless of the other policies. Therefore, the results from event-study analysis need to be interpreted very cautiously, even if the parallel trend assumption holds.

## Main Analysis: Dynamic Panel Regression Model

Motivated by the previously outlined limitations of the simple event-study design, we pursue an alternative dynamic panel data approach. We analyze the predictive effect of opening K–12 schools on case growth rates by panel data regression under the dynamic unconfoundedness assumption, where a specification is motivated by the susceptible-infectious-recovered-deceased (SIRD) model.

We emphasize at the outset and in *Limitations* that, while the motivation for this modeling is to approximate the causal effects of policies, holding other policies and other state variables fixed, the approach is based on observational (nonexperimental) data, and therefore the results should be interpreted with great caution. From a completely agnostic point of view, our results can be seen as uncovering predictive effects of in-person/hybrid school reopenings on case and death growth rates, controlling linearly for other policies, past infection levels, and county and state-week “fixed” effects. Furthermore, the actionable policies naturally supported from our predictive analysis—the staff masking and other mitigation measures in schools and prioritization of vaccination for teachers and elderly parents—appear to be nonharmful and have low implementation costs.

### Methods

*SI Appendix* provides the details for our research design, which closely follows that in ref. [11]. Fig. 4 is a causal path diagram [12, 13] for our model that describes how policies, behavior, and information interact together:

• The forward health outcome, $Y_{i,t+\ell }$, is determined last after all other variables have been determined.
• The policies, *P_it_*, affect health outcome $Y_{i,t+\ell }$ either directly or indirectly through mediators, human behavior *B_it_*, which may be only partially observed.
• Information variables, *I_it_*, such as lagged values of outcomes can affect human behavior and policies, as well as outcomes directly.
• The confounders *W_it_*, which vary across counties and time, affect all other variables; these include testing rates and unobserved but estimable county- and state-week effects.

The index *i* denotes the county *i*, and *t* and t+ℓ denote the time, where ℓ represents the time lag between infection and case confirmation or death. Our health outcomes are the growth rates in COVID-19 cases and deaths. Policy variables include school reopening in various modes, mask mandates, bans on gatherings, and stay-at-home orders, while information variables include lagged values of cases or deaths.

The causal structure allows for the effect of the policy to be either direct or indirect. For example, school openings not only directly affect case growth through the within-school transmission but also indirectly affect case growth by increasing parents’ mobility. The structure also allows for changes in behavior to be brought by the change in policies and information. The information variables, such as the actual recorded number of past confirmed cases, can cause people to spend more time at home, regardless of adopted policies; these changes in behavior, in turn, affect the transmission of SARS-CoV-2.

To further motivate our panel regression specification, we consider the SIRD model:

(2) $$\begin{array}{l} \dot{S}(t)=-\frac{S(t)}{N}\beta (t)I(t),\;\dot{I}(t)=\frac{S(t)}{N}\beta (t)I(t)-\gamma I(t),\\ \dot{R}(t)=(1-\kappa )\gamma I(t),\;\dot{D}(t)=\kappa \gamma I(t), \end{array}$$

where *S*, I, *R*, and *D* denote the number of susceptible, infected, recovered, and deceased individuals in a given state. Each of these variables is a function of time and dots indicate time derivative. *N* is the population, β(t) is the rate of infection spread, *γ* is the rate of recovery or death, and *κ* is the probability of death conditional on infection. Confirmed cases, *C*(*t*), evolve as

(3) $$\dot{C}(t)=\tau (t)I(t),$$

where τ(t) is the testing proportion (detection rate). Differentiating the logarithm of [**3**] and substituting [**2**], we have

(4) $$\frac{\ddot{C}(t)}{\dot{C}(t)}=\frac{S(t)}{N}\beta (t)-\gamma +\frac{\dot{\tau }(t)}{\tau (t)},$$

which indicates that the growth rate of cases depends on the rate of infection spread and the change in detection rates.

Our empirical specification is a discrete-time analog of Eq. **4** by approximating the case growth rate with the log difference in weekly confirmed cases and specifying $\frac{S(t)}{N}\beta (t)$ as a linear function of the variable for K–12 school visits, policies, past cases, county fixed effects, and state-week fixed effects:

(5) $$\begin{array}{l} \Delta_{7}\log\;\mathrm{Cas}e_{\text{it}}=\;\beta \prime \mathrm{Visi}t_{\text{i},\text{t}-14}+\sum_{\tau =14,21,28}\beta_{\text{y},\tau }\log\;\mathrm{Cas}e_{\text{i},\text{t}-\tau }\\ +\gamma \prime NPI_{\text{i},\text{t}-14}+\theta \mathrm{Tes}t_{\text{it}}+\alpha_{\text{i}}+\delta_{\text{s}(\text{i}),\text{w}(\text{t})}+\epsilon_{\text{it}}, \end{array}$$

where *i* is county, and *t* is day. The outcome variable $\Delta_{7}\log\;\mathrm{Cas}e_{\text{it}}:=\log\;\mathrm{Cas}e_{\text{it}}-\log\;\mathrm{Cas}e_{\text{i},\text{t}-7}$ is the log difference over 7 d in reported weekly cases with $\mathrm{Cas}e_{\text{it}}$ denoting the number of confirmed cases from day *t* – 6 to *t*. For the observation with zero weekly cases, we set the value of the log of weekly cases, $\log\;\mathrm{Cas}e_{\text{it}}$, to be –1.

$\mathrm{Visi}t_{\text{i},\text{t}-14}$ includes per-device K–12 school visits and college visits lagged by 14 d from the SafeGraph foot traffic data (*SI Appendix*, Fig. S4 *C* and *F*). The direct measure of K–12 school visits has advantages over that of school opening modes from the MCH data because the latter is prone to measurement errors caused by unrecorded changes in teaching methods and school closures beyond the information recorded in the MCH data. The measure of K–12 school visits may also capture student density heterogeneity within the same opening mode, especially for the hybrid teaching method.

As confounders, we consider a set of county fixed effects, *α_i_*, as well as state-week fixed effects, $\delta_{s(i),w(t)}$. County fixed effects *α_i_* control permanent differences across counties in unobserved personal risk aversion and attitude toward mask wearing, hand washings, and social distancing. The coefficient $\delta_{s(i),w(t)}$ on the interaction terms between state dummy variables and week dummy variables captures any change over time in people’s behaviors and NPIs that are common within a state; they also control for changes in weather, temperature, and humidity within a state. $NPI_{\text{i},\text{t}-14}$ includes county-level NPIs (mask mandates, banning gathering of more than 50 persons, stay-at-home orders) lagged by 2 wk that control for the effect of people’s behavioral changes driven by county-level policies on case growths. NPI data on stay-at-home orders and gathering bans is from Wu and coworkers [14] while the data on mask policies is from ref. [15]. These NPI data contain information up to the end of July; in our regression analysis, we set the value of these policy variables after August to be the same as the last day of observations.

$\mathrm{Tes}t_{\text{it}}$ is the growth rate of the number of tests recorded at the daily frequency for each state to capture the changes in detection rates $\dot{\tau }(t)/\tau (t)$ in [**4**]. This variable is important to fill the gap between confirmed cases and actual infections.

Finally, the logarithms of past weekly confirmed cases denoted by $\log\;\mathrm{Cas}e_{\text{i},\text{t}-\tau }$ for τ=14,21, and 28 are included in [**4**] as important confounders representing information variables. First, because the timing and the mode of school openings are likely to be affected by the number of lagged cases or deaths (e.g., the decision to reopen schools in California and Oregon depended on trends in local case counts) [16], controlling for the past weekly cases is critical for the unconfoundedness assumption.^¶^ Second, controlling for past confirmed cases is important because people may voluntarily change their risk-taking behavior in response to the new information provided by the confirmed cases rather than the actual, but unknown, number of infected individuals. On the other hand, people’s behavior may also be affected by the actual number of infected individuals beyond the reported cases, given that some people may see their friends or family get COVID-19.^ǁ^

We also consider an alternative specification using the proportion of K–12 students attending schools with teaching method p∈{in-person,hybrid,remote} constructed from the MCH data in place of the visits to K–12 schools from the foot traffic data. Furthermore, we investigate the role of mitigation strategies at school on the transmission of SARS-CoV-2 by examining how the coefficients of K–12 school visits and K–12 school openings depend on the mask-wearing requirement for staff with an interaction term between school opening variables and a dummy variable for staff mask-wearing requirements.

For parameter identification, we assume that the error *ϵ_it_* in [**5**] is orthogonal to the observed explanatory variables of school visits/openings, NPIs, and test rates and the past log cases, county fixed effects, and state-week fixed effects. The estimated parameters for school openings can be causally interpreted under the unconfoundedness assumption that the variables related to school openings (school visits, opening dates, teaching methods) are as good as randomly assigned after conditioning on other controls, county fixed effects, and state-week fixed effects.

Because the fixed effects estimator with a set of county dummies for dynamic panel regression could be severely biased when the time dimension is short compared to the cross-sectional dimension [17], we employ the debiased estimator by implementing bias correction [18] although the fixed effects estimator without bias correction gives qualitatively similar results. See *SI Appendix*, Table S5 and Fig. S8 for the results from the fixed effects estimator without bias correction.

### Result

Table 2 reports the debiased estimates of predictive effects of our dynamic panel data regression models. Clustered SEs at the state level are reported in parentheses to provide valid inference under possible dependency over time and across counties within each state. The results suggest that an increase in the visits to K–12 schools and opening K–12 schools with an in-person learning mode predict (are associated with) an increase in the growth rates of cases with 2 wk lag when schools implement no mask mandate for staff.

**Table 2:** Predictive effects (association) of school/college openings and other NPIs on case growth in the United States: debiased estimator

	Dependent variable: Case growth rates			
	1	2	3	4
K–12 visits,	0.467${}^{***}$	0.386${}^{***}$		
14-d lag	(0.070)	(0.070)		
K-12 visits		0.297${}^{***}$		
× 14-d lag		(0.070)		
no mask,				
K–12 in person,			0.047${}^{***}$	0.023
14-d lag			(0.017)	(0.021)
K–12 hybrid,			– 0.008	– 0.037${}^{***}$
14-d lag			(0.014)	(0.013)
K–12 remote,			– 0.082${}^{***}$	– 0.102${}^{***}$
14-d lag			(0.016)	(0.015)
K–12 in person				0.041${}^{**}$
× no mask,				(0.019)
14-d lag				
K–12 hybrid				0.049${}^{***}$
× no mask,				(0.017)
14-d lag				
College visits,	0.139${}^{*}$	0.070	0.132${}^{**}$	0.010
14-d lag	(0.071)	(0.073)	(0.064)	(0.076)
Mandatory	– 0.113${}^{***}$	– 0.123${}^{***}$	– 0.128${}^{***}$	– 0.128${}^{***}$
mask,	(0.018)	(0.017)	(0.020)	(0.019)
14-d lag				
Ban gatherings,	– 0.124${}^{***}$	– 0.136${}^{***}$	– 0.135${}^{***}$	– 0.137${}^{***}$
14-d lag	(0.033)	(0.044)	(0.033)	(0.042)
Stay at home,	– 0.264${}^{***}$	– 0.260${}^{***}$	– 0.261${}^{***}$	– 0.268${}^{***}$
14-d lag	(0.031)	(0.039)	(0.034)	(0.040)
Log(cases),	– 0.101${}^{***}$	– 0.101${}^{***}$	– 0.098${}^{***}$	– 0.099${}^{***}$
14-d lag	(0.009)	(0.010)	(0.010)	(0.010)
Log(cases),	– 0.061${}^{***}$	– 0.060${}^{***}$	– 0.060${}^{***}$	– 0.059${}^{***}$
21-d lag	(0.005)	(0.005)	(0.005)	(0.005)
Log(cases),	– 0.030${}^{***}$	– 0.033${}^{***}$	– 0.031${}^{***}$	– 0.034${}^{***}$
28-d lag	(0.003)	(0.003)	(0.004)	(0.004)
Test growth	0.009${}^{**}$	0.008${}^{*}$	0.009${}^{**}$	0.009${}^{**}$
rates	(0.004)	(0.004)	(0.004)	(0.004)
County	Yes	Yes	Yes	Yes
dummies				
State ×	Yes	Yes	Yes	Yes
week				
dummies				
Observations	690,297	545,131	612,963	528,941
R^2^	0.092	0.093	0.092	0.094

Dependent variable is the log difference over 7 d in weekly positive cases. Regressors are 7-d moving averages of corresponding daily variables and lagged by 2 wk to reflect the time between infection and case reporting except for test growth rates. All regression specifications include county fixed effects and state-week fixed effects. The debiased fixed effects estimator is applied. The results from the estimator without bias correction are presented in *SI Appendix*, Table S5. Asymptotic clustered SEs at the state level are reported in parentheses. ${}^{*}$ *P* < 0.1; ${}^{**}$ *P* < 0.05; ${}^{***}$ *P* < 0.01.

In Table 2, column 1, that of per-device visits to K–12 schools is 0.47 (SE = 0.07). The change in top 5 percentile values of per-device visits to K–12 schools between June and September among counties is around 0.15 in *SI Appendix*, Fig. S4*C*. Taking this value as a benchmark for full openings, fully opening K–12 schools may have contributed to (0.47 × 0.15 =) 7 percentage points increase in case growth rates. Table 2, column 3 indicates that openings of K–12 schools with the in-person mode are associated with 5 (SE = 2) percentage point increases in weekly case growth rates. It also provides evidence that openings of K–12 schools with remote learning mode are associated with lower case growth, perhaps because remote school opening induces more precautionary behavior to reduce transmission risk.

In Table 2, column 2, the estimated coefficient of the interaction between K–12 school visits and no mask-wearing requirements for staff is 0.24 (SE = 0.07), providing evidence that mask-wearing requirements for staff may have reduced the transmission of SARS-CoV-2 at schools. Similarly, in Table 2, column 4, the coefficients on the interaction of in-person and hybrid school openings with no mask mandates are positively estimated as 0.04 (SE = 0.02) and 0.05 (SE = 0.02), respectively. These estimates likely reflect not only the effect of mask-wearing requirements for staff but also that of other mitigation measures. For example, school districts with staff mask-wearing requirements frequently require students to wear masks and often increase online instructions.

Other studies on COVID-19 spread in schools have also pointed to the importance of mitigation measures. In contact tracing studies of cases in schools, ref. [19] found that six of seven traceable case clusters were related to clear noncompliance with mitigation protocols, and ref. [20] found that most secondary transmissions were related to absent face coverings. Ref. [21] found that children who tested positive for COVID-19 are considerably less likely to have reported consistent mask use by students and staff inside their school.

**Fig. 5:**
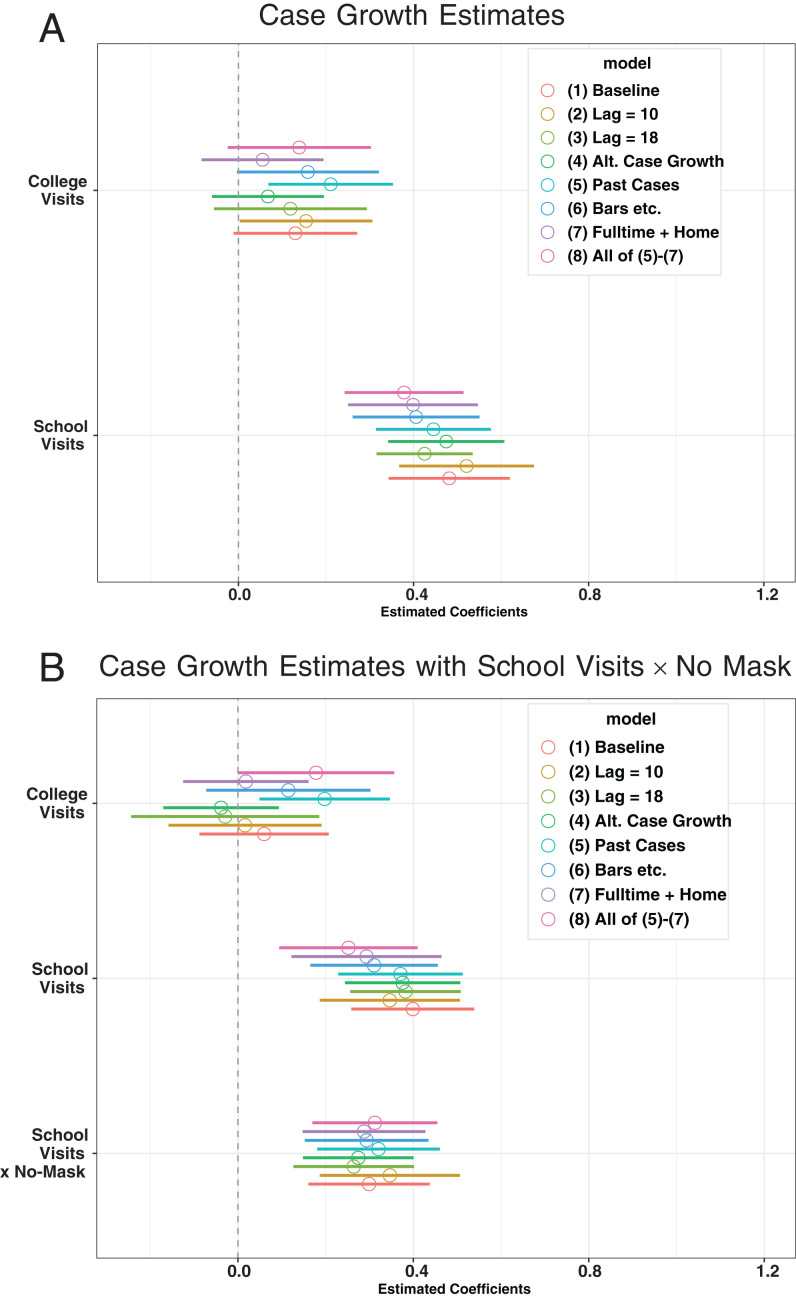
Sensitivity analysis for the estimated coefficients of K–12 visits and college visits of case growth regressions: debiased estimator. *A* presents the estimates of college visits and K–12 school visits with the 90% confidence intervals across different specifications taking Table 2, column 1 as baseline. *B* presents the estimates of college visits, K–12 school visits, and the interaction between K–12 school visits and no mask-wearing requirement for staff taking Table 2, column 2 as baseline. The results are based on the debiased estimator. *SI Appendix*, Fig. S8 presents the results based on the estimator without bias correction.

The estimated coefficient of per-device visits to colleges is 0.14 (SE = 0.07) in Table 2, column 1. With the change in top 5 percentile values of college visits between June and September as a benchmark for full openings, which is about 0.1, fully opening colleges may be associated with (0.14 × 0.1 =) 1.4 percentage points increase in case growth. Therefore, the estimated association of opening colleges with case growth is much smaller than that of opening K–12 schools. Furthermore, alternative specifications in Table 2, columns 2 and 4 and those in Fig. 5 yield smaller, and sometimes insignificant, estimates for college visits. This sensitivity may be due to the limited variation in changes in college visits over time across counties in the data, where the 75th percentile value of college visits is consistently very low (*SI Appendix*, Fig. S4*F*). *SI Appendix* presents more discussions on the association of opening colleges with the spread of COVID-19, where *SI Appendix*, Figs. S2 and S3 provide descriptive evidence that opening colleges and universities may be associated with the spread of COVID-19 in counties where large public universities are located.

Consistent with evidence from US state-level panel data analysis in ref. [11], the estimated coefficients of county-wide mask mandate policy are negative and significant in Table 2, columns 1 to 4, suggesting that mandating masks reduces case growth. The estimated coefficients of bans on gatherings and stay-at-home orders are also negative. The negative estimates of the log of past weekly cases are consistent with a hypothesis that the information on higher transmission risk induces people to take precautionary actions voluntarily to reduce case growth. Table 2 also highlights the importance of controlling for the test growth rates as a confounder.

Evidence on the role of schools in the spread of COVID-19 from other studies is mixed. Papers that focus on contract tracing of cases among students find limited spread from student infections ([19], [20], [22]– [25]). There is also some evidence that school openings are associated with increased cases in the surrounding community. Ref. [26] provides suggestive evidence that school openings are associated with increased cases in Montreal neighborhoods. Ref. [27] uses US state-level data to argue that school closures at the start of the pandemic substantially reduced infection.

Three closely related papers also examine the relationship between schools and county-level COVID-19 outcomes in the United States. Ref. [28] examines the relationship between schooling and cases in counties in Washington and Michigan. They find that in-person schooling is associated with increased cases only in areas with high preexisting COVID-19 cases. Similarly, ref. [29] analyzes US county-level data on COVID-19 hospitalizations and finds that in-person schooling is not associated with increased hospitalizations in counties with low preexisting COVID-19 hospitalization rates. The outcome variable of our regression analysis is case growth rates instead of new cases or hospitalizations. Consistent with refs. [28] and [29], our finding of a constant increase in growth rates implies a greater increase in cases in counties with more preexisting cases. Ref. [30] finds that counties with hybrid or remote openings had fewer cases than those with in-person openings but finds no association of teaching modes with deaths during the first 3 wk of the school year in Illinois. Our finding on death rates does not necessarily contradict that of ref. [30] because the 3-wk period is too short to identify the effect on deaths, whereas we examine the effect on deaths after 3 to 5 wk of openings.

We next provide sensitivity analysis by changing our regression specifications and assumptions about delays between infection and reporting cases as follows:

1) Baseline specifications in Table 2, columns 1 and 2.
2) and 3) Alternative time lags of 10 and 18 d for visits to colleges and K–12 schools as well as NPIs.
3) Setting the log of weekly cases to 0 when we observe zero weekly cases to compute the log difference in weekly cases for the outcome variable.
4) Add the log of weekly cases lagged by 5 wk and per-capita cumulative number of cases lagged by 2 wk as controls.
5) Add per-device visits to restaurants, bars, recreational places, and churches lagged by 2 and 4 wk as controls.
6) Add per-device visits to full-time and part-time workplaces and a proportion of devices staying at home lagged by 2 wk as controls.
7) All of 5) to 7).

Because the actual time lag between infection and reporting cases may be shorter or longer than 14 d, we consider the alternative time lags in specifications 2 and 3. Specification 4 checks the sensitivity of handling zero weekly cases to construct the outcome variable of the log difference in weekly cases.

A major concern for interpreting our estimate in Table 2 as the causal effect is that a choice of opening timing, teaching methods, and mask requirements may still reflect (residual, unaccounted) sources of confounding. Our baseline specification models are confounding by controlling for past infection rates (log of cases), other past implemented policies, and “latent” county- and state-week fixed effects. However, a choice of school openings may still be correlated with unmodeled time-varying unobserved factors at the county level. To further examine the sensitivity to the inclusion of potential other confounders, we estimate a specification with additional time-varying county-level controls in specifications 5 to 8.

Fig. 5*A* takes Table 2, column 1 as a baseline specification and plots the estimated coefficients for visits to colleges and K–12 schools with the 90% confidence intervals across different specifications using the debiased estimator; the estimates using the standard estimator without bias correction are qualitatively similar and reported in *SI Appendix*, Fig. S8. The estimated coefficients of K–12 school visits and college visits are all positive across different specifications, suggesting that an increase in visits to K–12 schools and colleges is robustly associated with higher case growth. On the other hand, the estimated coefficients often become smaller when we add more controls. In particular, relative to the baseline, adding full-time/part-time workplace visits and staying-home devices leads to somewhat smaller estimated coefficients for K–12 school and college visits, suggesting that opening K–12 schools and colleges is associated with people returning to work and/or going outside more frequently.

In Fig. 5*B*, the estimated interaction terms of K–12 school visits and no mask-wearing requirements for staff in Table 2, column 1 are all positive and significant, robustly indicating a possibility that a mask-wearing requirement for staff may have helped to reduce the transmission of SARS-CoV-2 at schools when K–12 schools opened with the in-person teaching method.

*SI Appendix*, Tables S6 and S7 provide further robustness checks, showing that the results are similar even when we use the log of weekly cases in place of the log difference of weekly cases as the outcome variable.

### Association between School Openings and Mobility

As highlighted by a modeling study for the United Kingdom [31], there are at least two reasons why opening K–12 schools in person may increase the spread of COVID-19. First, opening K–12 schools increases the number of contacts within schools, which may increase the risk of transmission among children, parents, education workers, and communities at large. Second, reopening K–12 schools allows parents to return to work and increase their mobility in general, which may contribute to the transmission of COVID-19 at schools and workplaces.

To give insight into the role of reopening K–12 schools for parents to return to work and to increase their mobility, we conduct panel data regression analysis by taking visits to full-time workplaces and a measure of staying-home devices as outcome variables and use a similar set of regressors as in Table 2 but without taking 2-wk time lags.

Table 3 shows how the proportion of devices at full-time workplaces and that of staying-home devices are associated with visits to K–12 schools as well as their in-person openings. In Table 3, columns 1 and 2, the estimated coefficients of per-device K–12 school visits and opening K–12 schools for full-time work outcome variables are positive and especially large for in-person K–12 school opening. Similarly, the estimates in Table 3, columns 3 and 4 suggest the negative association of per-device K–12 school visits and opening K–12 schools with the proportion of devices that do not leave their home. *SI Appendix*, Table S4 also provides evidence that the visits to restaurants and bars are positively associated with the visits to K–12 schools and colleges. This is consistent with a hypothesis that opening K–12 schools allows parents to return to work and spend more time outside. This result may also reflect education workers returning to work. In Table 3, columns 3 and 4, the positive coefficient of current and past log cases suggests that people voluntarily choose to stay home when the transmission risk is high.

**Table 3:** Predictive effects (association) of school/college openings on full-time workplace visits and staying home in the United States: standard fixed effects estimator without bias correction

	Dependent variable			
	Full time: 1	Full time: 2	Stay home: 3	Stay home: 4
K–12 school	0.085${}^{***}$		– 0.019	
visits	(0.006)		(0.026)	
Open K–12		0.999${}^{***}$		– 0.924${}^{**}$
in person		(0.125)		(0.382)
Open K–12		0.490${}^{***}$		– 0.127
hybrid		(0.051)		(0.186)
Open K–12		0.236${}^{***}$		– 0.271
remote		(0.048)		(0.307)
College	– 0.040${}^{***}$	– 0.046${}^{***}$	– 0.144${}^{***}$	– 0.148${}^{***}$
visits	(0.004)	(0.006)	(0.024)	(0.026)
Mandatory	– 0.057	– 0.141${}^{**}$	0.016	0.036
mask	(0.042)	(0.053)	(0.259)	(0.250)
Ban	0.060	0.075	0.436	0.351
gatherings	(0.047)	(0.051)	(0.560)	(0.520)
Stay at	– 0.060${}^{*}$	– 0.066${}^{*}$	2.793${}^{***}$	2.809${}^{***}$
home	(0.031)	(0.033)	(0.329)	(0.340)
Log(cases)	0.005	0.003	0.288${}^{***}$	0.282${}^{***}$
	(0.004)	(0.005)	(0.028)	(0.028)
Log(cases),	– 0.001	– 0.005${}^{*}$	0.209${}^{***}$	0.207${}^{***}$
7-d lag	(0.002)	(0.003)	(0.019)	(0.017)
Log(cases),	– 0.0005	– 0.003	0.098${}^{***}$	0.097${}^{***}$
14-d lag	(0.002)	(0.002)	(0.023)	(0.024)
Observations	670,895	595,872	670,895	595,872
R^2^	0.870	0.853	0.889	0.888

Dependent variables are full-time workplace visits and staying-home devices per residing device. All regression specifications include county fixed effects and state-week fixed effects. The standard fixed effects estimator without bias correction is used. Clustered SEs at the state level are reported in parentheses. ${}^{*}$ *P* < 0.1; ${}^{**}$ *P* < 0.05; ${}^{***}$ *P* < 0.01.

Table 4 presents regression analysis similar to that in Table 2 but including the proportion of devices at full-time/part-time workplaces and those at home as additional regressors, which corresponds to specification 7 in Fig. 5. The estimates indicate that the proportion of staying-home devices is negatively associated with the subsequent case growth, while the proportion of devices at full-time workplaces is positively associated with the case growth. Combined with the estimates in Table 3, these results suggest that school openings may have increased the transmission of SARS-CoV-2 by encouraging parents to return to work and to spend more time outside. This mechanism can partially explain the discrepancy between our findings and various studies that focus on cases among students. Contract tracing of cases in schools, such as in refs. [20] and [22]– [25], often finds limited direct spread among students. On the other hand, ref. [32] finds that parents and teachers of students in open schools experience increases in infection rates.

**Table 4:** Predictive effects (association) of school/college opening, full-time/part-time work, and staying home on case growth in the United States: debiased estimator

	Dependent variable: case growth rates			
	1	2	3	4
K–12 visits,	0.393${}^{***}$	0.283${}^{***}$		
14-d lag	(0.075)	(0.087)		
K–12 visits ×		0.287${}^{***}$		
no mask,		(0.071)		
14-d lag				
K–12 in person,			0.015	– 0.007
14-d lag			(0.016)	(0.020)
K–12 hybrid,			– 0.028${}^{**}$	– 0.055${}^{***}$
14-d lag			(0.013)	(0.013)
K–12 remote,			– 0.094${}^{***}$	– 0.115${}^{***}$
14-d lag			(0.015)	(0.014)
K–12 in person				0.034${}^{*}$
× no mask,				(0.020)
14-d lag				
K–12 hybrid ×				0.043${}^{***}$
no mask,				(0.017)
14-d lag				
Full-time	– 0.117	0.186	0.956${}^{**}$	0.967${}^{**}$
work device,	(0.417)	(0.490)	(0.384)	(0.436)
14-d lag				
Part-time	0.262	0.466	0.820${}^{***}$	0.915${}^{***}$
work device,	(0.259)	(0.305)	(0.276)	(0.309)
14-d lag				
Staying-home	– 0.290${}^{***}$	– 0.283${}^{***}$	– 0.352${}^{***}$	– 0.332${}^{***}$
device,	(0.057)	(0.069)	(0.061)	(0.067)
14-d lag				
Observations	690,297	545,131	612,963	528,941
R^2^	0.092	0.093	0.092	0.094

Dependent variable is the log difference over 7 d in weekly positive cases. All regression specifications include county fixed effects and state-week fixed effects; college visits; three NPIs; and 2-, 3-, and 4-wk lagged log of cases. See *SI Appendix*, Table S8 for the estimated coefficients for NPIs and the log of current and past cases. The debiased fixed effects estimator is applied. Asymptotic clustered SEs at the state level are reported in parentheses. ${}^{*}$ *P* < 0.1; ${}^{**}$ *P* < 0.05; ${}^{***}$ *P* < 0.01.

In Table 4, columns 1 and 2 the estimated coefficients on K–12 school visits remain positive and large even after controlling for the mobility measures of returning to work and being outside home, which are mediator variables to capture the indirect effect of school openings on case growth through its effect on mobility. The coefficient on K–12 school visits is ∼75% as large in Table 4 as in Table 2, suggesting that that within-school transmission may be the primary channel through which school openings affect the spread of COVID-19. Furthermore, the estimated coefficient of the interaction of K–12 school visits with the no-mask variable in Table 4, column 2 does not change after adding these measures of returning to work, indicating the importance of precautionary measures at schools even after conditioning parents’ going back to work.

### Death Growth Regression

We also analyze the effect of school openings on death growth by estimating

(6) $$\begin{array}{l} \Delta_{21}\log\;\mathrm{Deat}h_{\text{it}}=\beta \prime \mathrm{Visi}t_{\text{i},\text{t}-35}+\sum_{\tau =35,42,49}\beta_{\text{y},\tau }\log\;\mathrm{Deat}h_{\text{i},\text{t}-\tau }\\ \;\;\;+\gamma \prime NPI_{\text{i},\text{t}-35}+\alpha_{\text{i}}+\delta_{\text{s}(\text{i}),\text{w}(\text{t})}+\epsilon_{\text{it}}, \end{array}$$

where the outcome variable $\Delta_{21}\log\;\mathrm{Deat}h_{\text{it}}:=\log\;\mathrm{Deat}h_{\text{it}}-\log\;\mathrm{Deat}h_{\text{i},\text{t}-21}$ is the log difference over 21 d in reported weekly deaths with $\mathrm{Deat}h_{\text{it}}$ denoting the number of reported deaths from day *t* – 6 to *t*. The log of weekly deaths, $\log\;\mathrm{Deat}h_{\text{it}}$, is set to be –1 when we observe zero weekly deaths. We take the log difference over 21 d rather than 7 d for measuring death growth because the time lag between infection and death reporting is stochastic and spreads over at least 2 wk.^**^

The explanatory variables in [**6**] are lagged by 35 d to capture the time lag of infection and death reporting. Taking a longer time lag than 35 d may capture the effect of school openings on deaths through the secondary infection better (e.g., an infection from children to parents/grandparents); therefore, we also consider a lag length of 42 and 49 d.

**Fig. 6:**
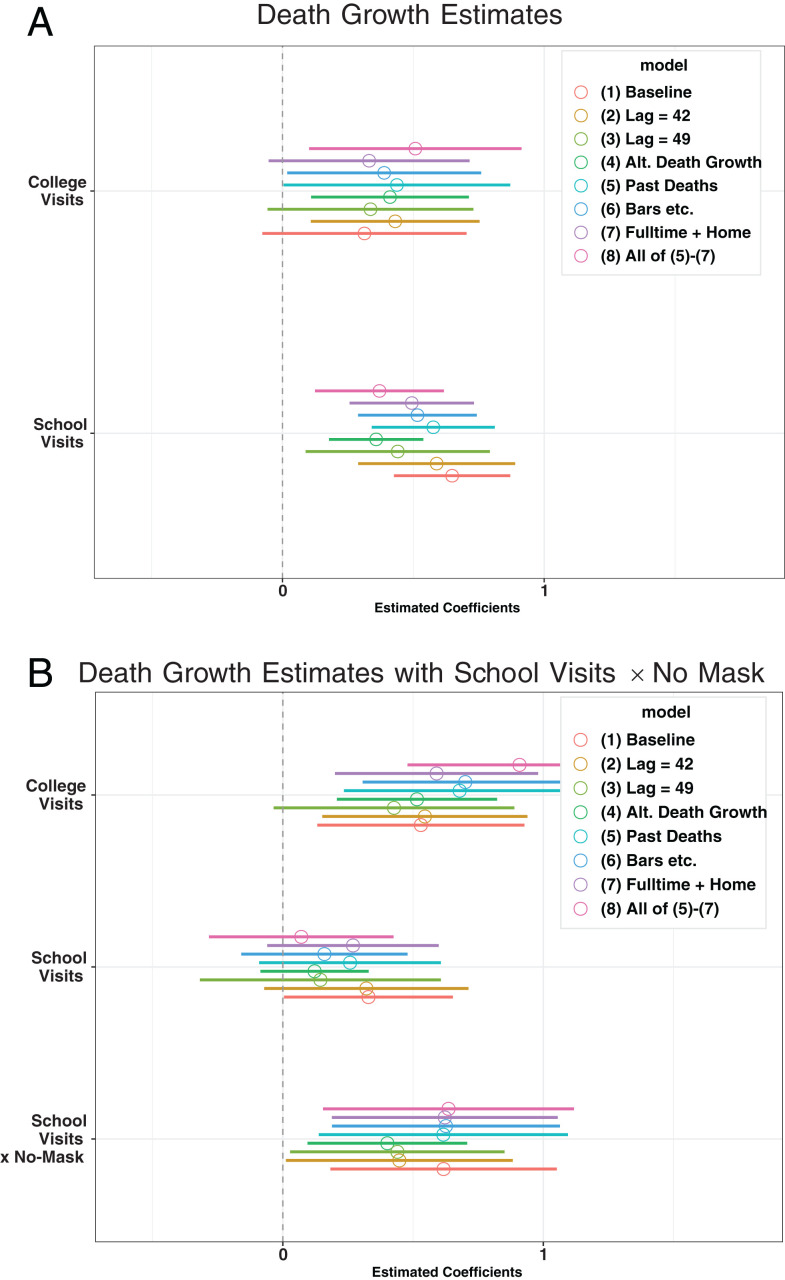
Sensitivity analysis for the estimated coefficients of K–12 visits and college visits of death growth regressions: debiased estimator. *A* presents the estimates of college visits and K–12 school visits with the 90% confidence intervals across different specifications taking *SI Appendix*, Table S9, column 1 as baseline. *B* presents the estimates of college visits, K–12 school visits, and the interaction between K–12 school visits and no mask-wearing requirement for staff taking *SI Appendix*, Table S9, column 2 as baseline.

Fig. 6 illustrates the estimated coefficients of visits to colleges and K–12 schools across different specifications for death growth regressions. Fig. 6*A* shows that the coefficients of visits to colleges and K–12 schools are positively estimated for 1) baseline, 2) and 3) an alternative time lag of 42 and 49 d, 4) setting the log of weekly deaths to 0 when we observe zero weekly deaths to compute death growth over 3 wk, and 5) to 8) adding more controls, providing robust evidence that an increase in visits to colleges and K–12 schools is positively associated with the subsequent increase in weekly death growth rates. Fig. 6*B* corresponds to Fig. 5*B*, showing that the association of K–12 school visits with death growth is stronger when no mask mandate for staff is in place.

*SI Appendix*, Table S9 reports the baseline estimates while *SI Appendix*, Table S10 shows those for the specification with staying-home devices and workplace visits. The estimated coefficients on K–12 school visits remain positive and large even after controlling for the variables of returning to work and being outside the home in *SI Appendix*, Table S10, suggesting the possible role of within-school transmission for a rise in deaths after school openings. These results are consistent with our findings in case growth regressions.

## Limitations

Our study has the following limitations. First, our study is observational and should be interpreted with great caution. Indeed, the potential presence of unobserved/unaccounted confounding factors can invalidate the interpretation of the predictive effects as causal effects. While we control for a variety of potential confounding factors, including other mitigation policies, past infection rates, and county- and state-week fixed effects, the decisions to open K–12 schools may be still correlated with other unobserved time-varying county-level factors that affect the spread of COVID-19. For example, people’s attitudes toward social distancing, hand washing, and mask wearing may change over time (which we cannot observe in the data). Their changes may be correlated with school opening decisions beyond the controls we added to our regression specifications.

Our analysis is also limited by the quality and the availability of the data as follows. The reported number of cases is likely to understate true COVID-19 incidence, especially among children and adolescents because they are less likely to be tested than adults given that children exhibit milder or no symptoms. This is consistent with Centers for Disease Control (CDC) data that show the lower testing volume and the higher rate of positive test among children and adolescents than among adults [9]. County-level testing data are not used because of a lack of data, although state-week fixed effects control for the weekly difference across counties within the same state and we also control daily state-level test growth rates.

Because foot traffic data are constructed from mobile phone location data, the data on K–12 school visits likely reflect the movements of parents and older children who are allowed to carry mobile phones to schools and exclude those of younger children who do not own mobile phones.^#^

Because COVID-19–infected children and adolescents are known to be less likely to be hospitalized or die from COVID-19, the consequence of transmission among children and adolescents driven by school openings crucially depends on whether the transmission of SARS-CoV-2 from infected children and adolescents to the older population can be prevented.^##^ Our analysis does not provide any empirical analysis on how school opening is associated with the transmission across different age groups due to data limitations.^###^ Ref. [32] shows that teachers in open schools experience higher COVID-19 infection rates compared to teachers in closed schools. They also show that this increase in infection rate also occurs in partners of teachers and parents of students in open schools.

The impact of school openings on the spread of COVID-19 on case growth may be different across counties and over time because it may depend not only on in-school mitigation measures but also on contact tracing, testing strategies, and the prevalence of community transmissions [16, 35]. We do not investigate how the association between school openings and case growths depends on contact tracing and testing strategies at the county level due to data limitation.

The result on the association between school opening and death growth in Fig. 6 is suggestive but must be viewed with caution. The time lag between infection and death is stochastic and spreads over time, making it challenging to uncover the relationship between the timing of school openings and subsequent deaths. Furthermore, while we provide sensitivity analysis for handling zero weekly deaths to approximate death growth, our construction of the death growth outcome variable remains somewhat arbitrary.

Finally, our result does not imply that K–12 schools should be closed. Closing schools can have negative impacts on children’s learning [36] and may cause declining physical and mental health among children and their parents (35–37). On the other hand, there is emerging evidence of long-term harm on children’s health induced by COVID-19 [40]. The decision to open or close K–12 schools requires careful assessments of the cost and the benefit by policymakers. However, given their relatively low implementation costs, our findings strongly support policies that enforce masking and other precautionary actions at school and prioritizing vaccines for education workers and elderly parents/grandparents.

## Data Availability

Anonymized comma-separated values data have been deposited in https://github.com/ubcecon/covid-schools.

## Footnotes

^*^MCH Strategic Data provides the school district-level data on whether each school district adopts the following mitigation strategies: 1) mask requirements for staff, 2) mask requirements for students, 3) prohibiting sports activities, and 4) online instruction increases, among other measures. We decided to use mask requirements for staff as the primary variable for the school mitigation strategy because it has a lower number of missing values than other mitigation measures.

^†^Our regression analysis uses 2,788 counties for specification with K–12 school opening with different teaching modes, while the sample contains 2,204 counties for specification with mask requirements for staff.

^‡^Although the workplace visits appear to start increasing before school opens in Fig. 1*D*, this reflects a measurement error about school opening dates as well as the use of the 7-d average visits. *SI Appendix*, Fig. S6*D* shows that a sharp increase in the daily visits to workplaces happens only on the day of school openings without any increase before for a subset of counties such that the school opening date is the same across all school districts within a county.

^§^As discussed in refs. [4] and [5], the canonical linear two-way fixed effects (TWFE) regressions with both time fixed effects and unit fixed effects may lead to a biased estimator for the “exposure” effect when exposure timings differ across units and, at the same time, the “exposure effects” evolve over time. The bias arises because the exposure effect estimate under TWFE regression partly relies on the wrong control units that have already been exposed to treatment under the staggered treatment design.

^¶^Referring to the causal path diagram in Fig. 4, the information variables affect both policies (e.g., school openings) and the people’s behavior (see ref. [11] and Table 3 for empirical evidence). Even if information variables do not affect outcome directly, they are important confounders because in the terminology of Pearl [12], they open a “backdoor” path from outcome to policy, which creates a noncausal association between policies and outcomes. By controlling for information variables (and other confounders), we block noncausal associations, revealing the association generated by the causal effect of policies on outcomes. See our discussion in *SI Appendix* as well as ref. [12] for more details.

^ǁ^Viewing the reported cases as a proxy for the actual number of infected individuals with measurement errors, if measurement errors at *t* – 7 and *t* – 14 are correlated, then such a correlation may bias the estimate given that the left-hand side variable contains $\log\;\mathrm{Cas}e_{\text{i},\text{t}-7}$ while the right-hand side includes $\log\;\mathrm{Cas}e_{\text{i},\text{t}-14}$ in [**5**].

^**^For deceased persons above 65 y old between 1 March 2020 and 31 January 2021, the CDC estimates that the interquartile range for the number of days from symptom onset to death is (9, 25) d while the interquartile range from death to reporting is (4, 59) d. See table 2 of https://www.cdc.gov/coronavirus/2019-ncov/hcp/planning-scenarios.html.

^#^We also focus on limited points of interest: K–12 schools, colleges and universities, restaurants, drinking places, other recreational places including recreational facilities, and churches. We check the robustness by including visits to assisted-living facilities for the elderly and nursing care facilities as additional controls, but the results are not sensitive to their inclusion.

^##^In the meta-analysis of 54 studies on the household transmission of SARS-CoV-2 [33], estimated household secondary attack rate to child contacts was 16.8%. Ref. [34] reports that household secondary attack rate from children and adolescents to other family members was 23.8% and higher than other age groups in Japan.

^###^The CDC collects the data on the number of reported cases by age groups from each state whenever such data are available. However, for many counties, the reported cases by age groups are missing or there exists a substantial gap between the sum of cases across different age groups reported by the CDC and the total number of cases reported in NYT case data (see, for example, the case of Ingham, MI, in *SI Appendix*, Fig. S3).
